# Post-radioembolization yttrium-90 PET/CT - part 2: dose-response and tumor predictive dosimetry for resin microspheres

**DOI:** 10.1186/2191-219X-3-57

**Published:** 2013-07-25

**Authors:** Yung-Hsiang Kao, Jeffrey D Steinberg, Young-Soon Tay, Gabriel KY Lim, Jianhua Yan, David W Townsend, Charley A Budgeon, Jan A Boucek, Roslyn J Francis, Timothy ST Cheo, Mark C Burgmans, Farah G Irani, Richard HG Lo, Kiang-Hiong Tay, Bien-Soo Tan, Pierce KH Chow, Somanesan Satchithanantham, Andrew EH Tan, David CE Ng, Anthony SW Goh

**Affiliations:** 1 Department of Nuclear Medicine and PET, Singapore General Hospital, Outram Road, Singapore 169608, Singapore; 2 Department of Nuclear Medicine, Sir Charles Gairdner Hospital, Hospital Ave, Perth, Western Australia 6009, Australia; 3 Department of Nuclear Medicine, Austin Hospital, Level 1, Harold Stokes Building, 145 Studley Rd, Heidelberg, Melbourne, Victoria 3084, Australia; 4 Singapore Bioimaging Consortium, Agency for Science, Technology and Research (A*STAR), 11 Biopolis Way, Helios Building, Singapore 138667, Singapore; 5 Agency for Science, Technology and Research (A*STAR) - National University of Singapore, Centre for Life Sciences, National University of Singapore (NUS) Clinical Imaging Research Centre, 14 Medical Drive, Singapore 117599, Singapore; 6 Centre for Applied Statistics, University of Western Australia, 35 Stirling Highway, Perth, Western Australia 6009, Australia; 7 Department of Research, Sir Charles Gairdner Hospital, Hospital Ave, Perth, Western Australia 6009, Australia; 8 Department of Radiation Oncology, National University Cancer Institute Singapore (NCIS), 1E Kent Ridge Road, NUHS Tower Block, Level 7, Singapore 119228, Singapore; 9 Department of Diagnostic Radiology, Singapore General Hospital, Outram Road, Singapore 169608, Singapore; 10 Department of General Surgery, Singapore General Hospital, Outram Road, Singapore 169608, Singapore; 11 Department of Surgical Oncology, National Cancer Centre Singapore, 11 Hospital Drive, Singapore 169610, Singapore; 12 Office of Clinical Sciences, Duke-National University of Singapore Graduate Medical School, 8 College Rd, Singapore 169857, Singapore; 13 School of Medicine and Pharmacology, University of Western Australia, 35 Stirling Highway, Perth, Western Australia 6009, Australia; 14 Department of Radiology, Leiden University Medical Center, Albinusdreef 2, 2300 RC Leiden, The Netherlands

**Keywords:** Yttrium-90 radioembolization, Selective internal radiation therapy, PET/CT, Voxel dosimetry, Dose-volume histogram, Predictive dosimetry

## Abstract

**Background:**

Coincidence imaging of low-abundance yttrium-90 (^90^Y) internal pair production by positron emission tomography with integrated computed tomography (PET/CT) achieves high-resolution imaging of post-radioembolization microsphere biodistribution. Part 2 analyzes tumor and non-target tissue dose-response by ^90^Y PET quantification and evaluates the accuracy of tumor ^99m^Tc macroaggregated albumin (MAA) single-photon emission computed tomography with integrated CT (SPECT/CT) predictive dosimetry.

**Methods:**

Retrospective dose quantification of ^90^Y resin microspheres was performed on the same 23-patient data set in part 1. Phantom studies were performed to assure quantitative accuracy of our time-of-flight lutetium-yttrium-oxyorthosilicate system. Dose-responses were analyzed using ^90^Y dose-volume histograms (DVHs) by PET voxel dosimetry or mean absorbed doses by Medical Internal Radiation Dose macrodosimetry, correlated to follow-up imaging or clinical findings. Intended tumor mean doses by predictive dosimetry were compared to doses by ^90^Y PET.

**Results:**

Phantom studies demonstrated near-perfect detector linearity and high tumor quantitative accuracy. For hepatocellular carcinomas, complete responses were generally achieved at *D*_70_ > 100 Gy (*D*_70_, minimum dose to 70% tumor volume), whereas incomplete responses were generally at *D*_70_ < 100 Gy; smaller tumors (<80 cm^3^) achieved *D*_70_ > 100 Gy more easily than larger tumors. There was complete response in a cholangiocarcinoma at *D*_70_ 90 Gy and partial response in an adrenal gastrointestinal stromal tumor metastasis at *D*_70_ 53 Gy. In two patients, a mean dose of 18 Gy to the stomach was asymptomatic, 49 Gy caused gastritis, 65 Gy caused ulceration, and 53 Gy caused duodenitis. In one patient, a bilateral kidney mean dose of 9 Gy (*V*_20_ 8%) did not cause clinically relevant nephrotoxicity. Under near-ideal dosimetric conditions, there was excellent correlation between intended tumor mean doses by predictive dosimetry and those by ^90^Y PET, with a low median relative error of +3.8% (95% confidence interval, -1.2% to +13.2%).

**Conclusions:**

Tumor and non-target tissue absorbed dose quantification by ^90^Y PET is accurate and yields radiobiologically meaningful dose-response information to guide adjuvant or mitigative action. Tumor ^99m^Tc MAA SPECT/CT predictive dosimetry is feasible. ^90^Y DVHs may guide future techniques in predictive dosimetry.

## Background

Coincidence imaging of low-abundance yttrium-90 (^90^Y) internal pair production by ^90^Y positron emission tomography with integrated computed tomography (PET/CT) achieves high-resolution imaging of post-radioembolization microsphere biodistribution. In part 1, we reviewed the recent literature supporting the use of post-radioembolization ^90^Y PET/CT, described our scan protocol, patient cohort, diagnostic reporting guidelines, and results of qualitative analysis [1]. In brief, we showed that with proper diagnostic reporting technique and emphasis on continuity of care, the presence of background noise did not pose a problem and ^90^Y PET/CT consistently out-performed ^90^Y bremsstrahlung single-photon emission computed tomography with integrated CT (SPECT/CT) in all aspects of qualitative analysis [1].

The terms ‘predictive dosimetry’, ‘target’, ‘non-target’, ‘technical success’, and the ‘planning-therapy continuum’ were also defined in part 1 [1]. It is important for the understanding of part 2 to reiterate that our definition of technical success, an adaptation from conventional reporting standards [2], refers to the *qualitative* assessment of a satisfactory ^90^Y activity biodistribution in accordance with radiation planning expectations and should not be confused with ‘clinical success’ [2] which is *quantitatively* related to dose-response radiobiology.

A knowledge gap exists today between institutions which practice predictive dosimetry and others which rely on semi-empirical methods [3]. Readers who are accustomed to semi-empirical therapy planning may struggle to understand the dosimetric concepts discussed in this paper or its relevance to clinical practice. These readers are encouraged to refer to our recent series of publications explaining concepts in modern predictive dosimetry for ^90^Y resin microspheres and common misconceptions [3-6].

Where prognostication is of concern, qualitative analysis alone will not suffice [3]. The scientific language of dose-response radiobiology is the radiation absorbed dose expressed in grays (Gy), not the prescribed activity expressed in becquerels (Bq). To realize the full potential of post-radioembolization ^90^Y PET/CT, absorbed dose quantification should be performed in relevant clinical settings. As ^90^Y radioembolization is a brachytherapy delivered by β^-^-emitting microspheres, any tissue response is expected to follow predictable dose-response radiobiology. In principle, accurate knowledge of radiation thresholds in both target and non-target tissue facilitates the optimization of predictive dosimetry and enables the prognostication of technically unsuccessful cases to guide adjuvant therapy or mitigative action to minimize non-target radiation toxicity.

However, accurate tissue radiation thresholds for ^90^Y resin microspheres have remained elusive despite more than two decades of clinical use. Current radiation planning limits are broad and quote mean absorbed doses which falsely assume a uniform dose distribution: tumor > 120 Gy, non-tumorous liver < 50 to 70 Gy, lungs < 20 to 30 Gy [7-9]. As a consequence of this uncertainty, dosimetric dilemmas may be encountered in patients who may benefit from radiation planning up to the limits of normal tissue radiation tolerance. Furthermore, normal tissue radiation thresholds for ^90^Y resin microspheres shunted to non-target viscera such as the stomach or duodenum are largely unknown, precluding informed decision making for appropriate mitigative action. Historically, research into dose-response data had been technically challenging: intraoperative beta probes or histological examinations are invasive [10,11], quantification by ^90^Y bremsstrahlung scintigraphy is problematic and largely inaccurate [1], and predictive dosimetry simulated by ^99m^Tc macroaggregated albumin (MAA) is subject to variable accuracy due to the physical limitations of MAA [12].

Dosimetric studies have shown ^99m^Tc MAA to be feasible for simulating the post-radioembolization biodistribution of ^90^Y resin microspheres [13-16]. However, ^99m^Tc MAA is an imperfect surrogate for ^90^Y resin microspheres. Due to biophysical and technical differences such as particle size, specific gravity, injected particle load, microembolization, and catheter placement, ^99m^Tc MAA can never exactly replicate the post-radioembolization biodistribution of ^90^Y resin microspheres [12,17]. Therefore, predictive dosimetry simulated by ^99m^Tc MAA provides only an *estimate* of the tissue absorbed doses *intended* by the nuclear medicine physician [6]. Traditionally based on planar scintigraphy [13,14], modern predictive dosimetry employs SPECT/CT to tomographically assess the biodistribution of ^99m^Tc MAA and to improve its quantitative accuracy [6]. So far, the accuracy of ^99m^Tc MAA SPECT/CT predictive dosimetry has only been indirectly validated by inference from follow-up response [6,18,19]. For technically successful cases without visually significant discordant biodistribution between ^99m^Tc MAA and ^90^Y resin microspheres, a direct ‘Gy-to-Gy’ comparison of intended doses by ^99m^Tc MAA SPECT/CT predictive dosimetry versus post-radioembolization doses by microsphere biodistribution analysis has not yet been performed to date.

Recent experimental and clinical studies have shown post-radioembolization ^90^Y PET quantification to be accurate and feasible [1,20,21]. ^90^Y PET/CT thus presents a new opportunity to study the radiobiology of ^90^Y resin microspheres in a rapid, convenient, and noninvasive manner, with the ability to tomographically evaluate the dose distribution of an entire target organ in high resolution by a single scan. Moreover, quantitative ^90^Y PET data can be translated into dose-volume histograms (DVHs) to dosimetrically account for the heterogeneous nature of microsphere biodistribution.

In part 2 of our two-part retrospective report, we focus on post-radioembolization ^90^Y PET quantification on the same patient cohort as was reported in part 1 [1]. We analyze dose-responses in tumor and non-target tissue using ^90^Y PET-based voxel dosimetry or Medical Internal Radiation Dose (MIRD) macrodosimetry [7,22]. We describe the potential of ^90^Y DVHs to guide predictive dosimetry and discuss how the quantification of non-target absorbed doses may impact post-radioembolization care. Finally, we evaluate the accuracy of tumor ^99m^Tc MAA SPECT/CT predictive dosimetry in direct comparison to post-radioembolization doses by ^90^Y PET.

## Methods

### Phantom quantification

An institutional review board approval was obtained for the conduct and publication of this retrospective report (CIRB 2010/781/C, SingHealth, Singapore). Verbal consent was obtained from patients for scans to be performed. Quantitative phantom studies were first performed to confirm detector linearity of our time-of-flight lutetium-yttrium-oxyorthosilicate (LYSO) system and for comparison with a radiopharmacy dose calibrator. ^90^Y resin microspheres (SIR-Spheres, Sirtex Medical Limited, North Sydney, Australia) were used. We scanned the microsphere sediment and not its fresh suspension to avoid issues with microsphere sedimentation during PET acquisition. In effect, each vial sediment represents a small heterogeneous volume of high ^90^Y radioconcentration, simulating a small tumor implanted with a high and heterogeneous density of ^90^Y resin microspheres. All microsphere sediments were scanned within its original glass shipping vial for convenience and to avoid operator radiation exposure for microsphere extraction.

**Figure 1:**
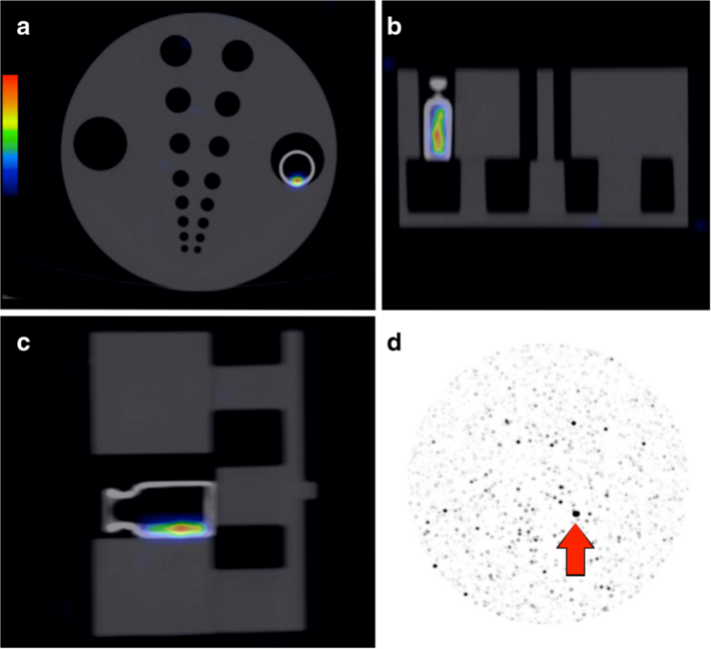
**Example of a **^**90**^**Y PET/CT phantom scan with maximum intensity projection image. (a)** Trans-axial, **(b)** coronal, and **(c)** sagittal planes. This scan shows our lowest tested vial sediment activity of 3.5 MBq (≈3 MBq/ml). The upper PET visual display threshold of this scan was adjusted to 114% (2,000 kBq/ml) to minimize visual interference from background noise [1]. **(d)** Maximum intensity projection image of the same phantom scan (arrow indicates vial sediment) with the upper PET visual display threshold adjusted to 1% (20 kBq/ml) to reveal the underlying background noise as an example to readers [1].

Vials of microspheres were obtained by convenience sampling, either before or after extraction of the desired treatment activity. Phantom scans of high activities (>3.0 GBq) were performed on fresh vials on the day it was received from the manufacturer, while scans of very low activities (<0.1 GBq) were performed after a period of decay of residual vial activities. The vials were placed within a perspex body phantom and scanned in one bed position over 15 min (Figure 1). All scan parameters were identical to our clinical scan protocol described in part 1 [1].

For vial activity quantification, a volume of interest (VOI) encompassing the entire vial sediment was generated by selecting a fixed volumetric isocontour threshold of 1%. The total ^90^Y activity (kBq) within the VOI was calculated as the product of its mean radioconcentration (kBq/ml) and its volume (cm^3^). Each vial sediment quantified by ^90^Y PET was compared to that measured by a dose calibrator (Biodex Atomlab 500, Biodex Medical Systems, New York, NY, USA). Its purpose was to evaluate the general relative accuracy of ^90^Y PET quantification in the absence of a true reference standard and also to empirically assess quantitative effects from background noise, variable vial sediment volume, radioconcentration, vial geometry, or positron contributions from possible radionuclidic impurities such as yttrium-88 [23].

### Tissue quantification

Retrospective absorbed dose quantification by ^90^Y PET was performed on the same 23-patient data set as was reported in part 1 [1]. The two objectives were, first, to establish dose-response observations in tumor and non-target tissue and, second, to evaluate the accuracy of tumor ^99m^Tc MAA SPECT/CT predictive dosimetry [6] in direct Gy-to-Gy comparison to post-radioembolization doses by ^90^Y PET.

Tumors were analyzed using cumulative ^90^Y DVHs generated by voxel dosimetry. This was performed on a small select group of tumors treated under highly favorable, near-ideal dosimetric conditions to ensure reliable dose-response observations and to assess the best possible accuracy of tumor ^99m^Tc MAA SPECT/CT predictive dosimetry. The tumor selection criteria were as follows: technically successful ^90^Y radioembolization, well-circumscribed tumors with good overall activity coverage, mean tumor-to-normal liver (T/N) ratio ≥2 as estimated by ^99m^Tc MAA SPECT/CT, and follow-up diagnostic sectional imaging available for clinical correlation. All tumor VOIs were manually contoured slice by slice according to CT anatomical margins. We avoided using tumor VOIs generated by PET volumetric isocontour thresholding as these often do not represent their true anatomical extent due to heterogeneous microsphere biodistribution. Regions of absent ^90^Y activity in tumors were visually correlated to CT findings and excluded from VOI analysis if necrotic.

^90^Y PET voxel dosimetry was performed by a novel program written in Interactive Data Language (IDL 6.1), freely available from the corresponding author for research purposes [24]. This program was initially developed for total glycolytic volume analysis of ^18^F-fluorodeoxyglucose PET data [25], subsequently adapted for ^90^Y PET voxel dosimetry in this study. ^90^Y absorbed dose distributions were calculated using a simplified approach based on voxel mean radioconcentrations instead of dose kernel or Monte Carlo methods. Its dosimetric rationale and evidence to support this approach are detailed in Additional file 1.

The reconstructed ^90^Y PET voxels have a spatial resolution of approximately 10 to 12 mm at full width at half maximum. ^90^Y radiation cross fire and beta dose spread were taken to be similar to the blurring of activity due to the partial volume effect of PET. Although the dose contribution by ^90^Y bremsstrahlung may be assumed negligible within areas of ^90^Y activity [26], we accounted for such bremsstrahlung losses in our voxel dosimetry by adjusting our absorbed dose coefficient by a factor of 0.995 based on Monte Carlo calculations. ^90^Y dosimetric parameters used were as follows: half-life, 64.1 hours; energy per decay, 0.934 MeV [27]; absorbed dose coefficient, 49.59 Gy/GBq/kg; and soft tissue density, 1.04 g/cm^3^. The dosimetry assumes no post-implantation redistribution of microspheres, leaching, nor destruction of microspheres *in vivo*. Dose distribution heterogeneity was limited to the size of the reconstructed voxels; true dose heterogeneity at the microscopic level was not assessed.

Mean absorbed doses in tumor vascular thrombosis and gastric and duodenal walls were estimated by ^90^Y MIRD macrodosimetry [7,22]. For these small or thin-walled structures, ^90^Y DVH analysis was not feasible due to partial volume effect and uncertainties in manual VOI delineation on CT. These issues especially affect hollow viscus, such as the stomach and duodenum, as variable wall distension or compression introduces further uncertainties in manual VOI delineation. Instead, we chose to generate VOIs by PET volumetric isocontour thresholding, visually constrained to CT anatomical margins. This obtains the mean VOI radioconcentration, from which the VOI-specific mean absorbed doses were approximated by MIRD formularism. dose-response analysis was strictly limited to observations within these VOIs.

All tissue absorbed doses were decay-corrected back to the time of ^90^Y radioembolization. Tumor response was reported using the lesion-specific modified Response Evaluation Criteria in Solid Tumors [28]. In addition, we defined a ‘minor response’ as any tumor size reduction which does not fulfill the criteria for a partial response. As there is no current standard for ^90^Y DVH reporting, we selected the *D*_70_ and *V*_100_ values for tumor reporting, where *D*_70_ represents the minimum absorbed dose delivered to 70% of the tumor volume, and *V*_100_ represents the percentage tumor volume receiving ≥100 Gy. Non-target absorbed doses were expressed in accordance to external beam radiotherapy convention as far as possible [29,30]. Cases of non-target activity were followed up by retrospective review of clinical records, and relevant clinical toxicities were graded according to the Common Terminology Criteria for Adverse Events version 4.03 (CTCAE; National Institutes of Health, Bethesda, MD, USA).

The second objective of retrospective absorbed dose quantification was to evaluate the accuracy of tumor predictive dosimetry simulated by ^99m^Tc MAA SPECT/CT. This was performed by a direct, i.e., Gy-to-Gy, comparison between intended tumor mean doses and post-radioembolization tumor mean doses by ^90^Y PET, expressed as relative percentage errors. Our protocol for predictive dosimetry was previously described [6], which involves catheter-directed CT angiography for arterial territory delineation, quantitative ^99m^Tc MAA SPECT/CT of the target organ, and MIRD macrodosimetry to achieve artery-specific personalized radiation planning for ^90^Y resin microspheres.

### Statistical analysis

Data are presented as median, mean, range, and 95% confidence intervals (CI), where applicable. Linear regression with Pearson’s correlation coefficient (*r*) and Bland-Altman plots with intraclass correlation coefficients (ICC) were used for comparison of quantitative data; a value of ≥0.8 indicated excellent correlation.

## Results

### Phantom validation

**Figure 2:**
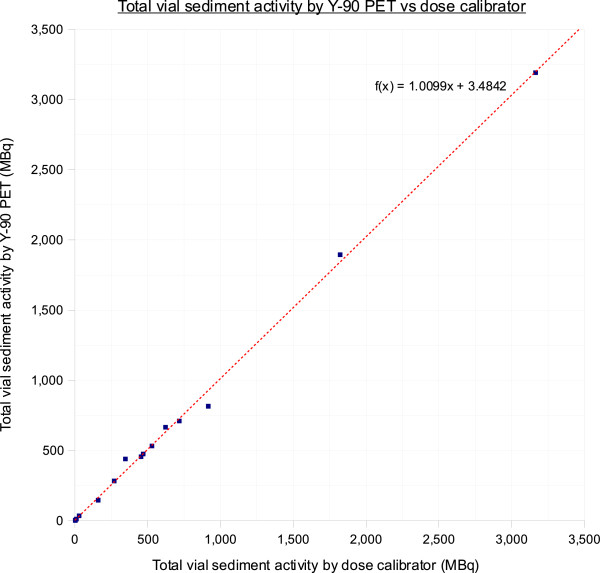
**Detector linearity.** Linear regression line (dotted) of total vial sediment activities quantified by ^90^Y PET versus that of a dose calibrator.

A total of 15 phantom scans were performed. Near-perfect detector linearity was demonstrated on our time-of-flight LYSO system within the tested limits of 3.5 MBq to 3.2 GBq (*r* = 0.99, calibration factor 0.99, Figure 2). Detector saturation was not a problem without ^90^Y bremsstrahlung shielding. The Bland-Altman plot showed excellent agreement between ^90^Y PET and dose calibrator measurements (ICC = 99.85%; Additional file 1: Figure S1).

Across all data points, the median relative quantification error of ^90^Y PET was +1.38% (mean +2.18%, 95% CI -3.92% to +8.28%). Vial activities >400 MBq had very low relative quantification errors (mean +0.29%, median +0.72%, 95% CI -3.30% to +3.87%), whereas vial activities <400 MBq had larger errors (mean +4.35%, median +4.95%, 95% CI -8.43% to +17.12%; Additional file 1: Figure S2) - a finding attributable to background noise from ^176^Lu within the LYSO crystal [31,32]. Based on these results, we deemed it feasible to assume all tumor activities quantified by ^90^Y PET to have negligible error for the purposes of this study.

### ^90^Y dose-response

**Figure 3:**
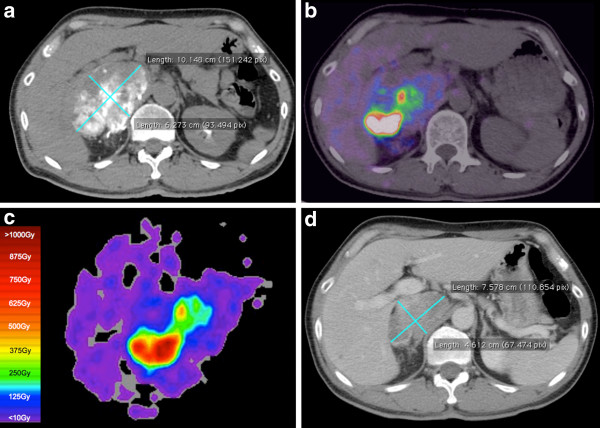
**Patient 9. **^90^Y radioembolization of an organ other than the liver. KIT-negative GIST with bulky metastasis to the right adrenal gland, refractory to tyrosine kinase inhibitors. Angiography was previously shown in part 1 [1]. **(a)** Catheter-directed CT angiogram of the right inferior adrenal artery delineates the targeted right adrenal tumor measuring 10.1 × 6.3 cm. **(b)** Post-radioembolization ^90^Y PET/CT depicts microsphere biodistribution in high resolution, with concordant activity within targeted regions of contrast-enhancing tumor. Low-grade activity seen in the adjacent right liver lobe is due to ^90^Y radioembolization of a segment IV metastasis. **(c)** Isodose map by voxel dosimetry of the corresponding trans-axial slice of the right adrenal tumor provides a visual representation of dose heterogeneity within the tumor and adjacent right liver lobe and displays the full dose range from 0 to >1,000 Gy. **(d)** Follow-up contrast-enhanced CT of the abdomen at 9.5 months shows a moderate size reduction to 7.6 × 4.6 cm, representing a partial response.

**Figure 4:**
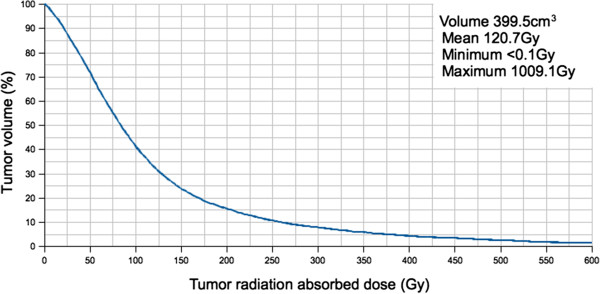
^**90**^**Y DVH of the right adrenal gland GIST metastasis generated by **^**90**^**Y PET voxel dosimetry.** Continued from Figure 3. A partial response was achieved at *D*_70_ 53 Gy.

**Figure 5:**
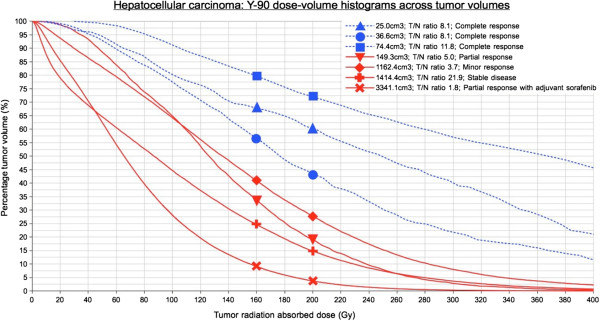
^**90**^**Y DVH of seven HCC tumors, ranging in size from 25.0 to 3,341.1 cm**^**3**^**.**

**Figure 6:**
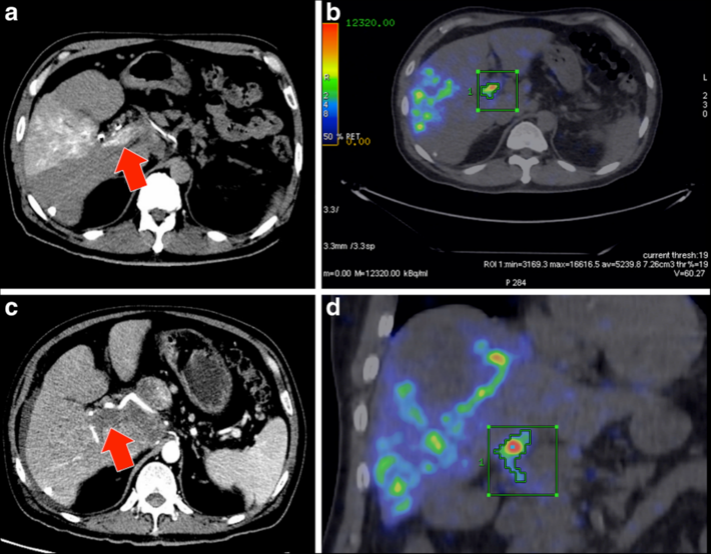
**Patient 10. (a)** Catheter-directed CT angiogram of the anterior branch of the right hepatic artery, supplying segments V and VIII, demonstrates contrast enhancement of a large portal vein tumor thrombus (arrow). **(b, c)** A ^90^Y PET VOI of the activity within the portal vein tumor thrombus was defined by volumetric isocontour thresholding, visually constrained to its anatomical margins as seen on the catheter-directed CT angiography. Within this VOI, the mean ^90^Y radioconcentration at the time of scan (5,239.8 kBq/ml) was decay-corrected back to the time of ^90^Y radioembolization (6,653 kBq/ml). By ^90^Y MIRD macrodosimetry, the mean absorbed dose of the portal vein tumor thrombus was approximately 316 Gy within the VOI. **(d)** Follow-up triphasic CT of the liver in the arterial phase at 4 months post-radioembolization demonstrates a complete lack of contrast enhancement within the VOI (arrow), suggesting a complete response and clinically validates the ^90^Y PET quantification.

Twenty-three post-radioembolization ^90^Y PET/CT scans were eligible for dose-response analysis, as was reported in part 1 [1]. As per our institutional protocol, the catheter tip for ^90^Y resin microsphere injection was placed in exactly the same position as for ^99m^Tc MAA injection in all cases, as visually assessed by the procedural interventional radiologist during ^90^Y radioembolization. Only eight patients fulfilled all tumor selection criteria: patients 1, 2, 8, 9, 17, 19, 21, and 23. The median tumor-to-normal liver (T/N) ratio estimated by ^99m^Tc MAA SPECT/CT was 5.0 (mean 8.0, range 1.8 to 21.9). Tumor ^90^Y DVH dose-response results involving hepatocellular carcinomas (HCC), cholangiocarcinoma, and adrenal metastatic gastrointestinal stromal tumor (GIST; Figures 3 and 4) are presented in Table 1. Across HCCs of various lesion sizes, cross-analysis of seven ^90^Y DVHs suggest that complete responses were generally achieved at *D*_70_ > 100 Gy, whereas tumors with incomplete responses generally have *D*_70_ < 100 Gy (Figure 5). Smaller HCCs, such as those <80 cm^3^, achieved *D*_70_ > 100 Gy more easily than larger tumors. dose-response results of portal vein tumor thrombosis by ^90^Y MIRD macrodosimetry (Figure 6) are presented in Table 2.

**Table 1:** **Tumor **^**90**^**Y dose-response by dose-volume histograms**

**Patient number**	**Tumor type**	**Tumor size (cm**^**3**^**)**	***D***_**70 **_**(Gy)**	***V***_**100 **_**(%)**	**Mean dose (Gy)**	**Follow-up (months)**	**Response**
23	HCC	≈2.4^a^	≈74^a^	≈47^a^	≈108^a^	3	Complete
8	HCC	≈4.7^a^	≈101^a^	≈71^a^	≈168^a^	2.8	Complete
23	HCC	25.0	142	80	263	3	Complete
23	HCC	36.6	122	77	215	3	Complete
21	HCC	74.4	213	91	425	5.5	Complete
23	HCC	149.3	89	65	137	3	Partial
1	HCC	1,162.3	85	64	151	1.8	Minor
2	HCC	1,414.4	37	45	105	6.7	Stable
19	HCC	3,341.1	40	28	77	12	Partial; adjuvant sorafenib
17	Cholangiocarcinoma	345.9	90	63	120	3.3	Complete
9	Adrenal GIST	399.5	53	41	121	9.5	Partial

^a^Subject to partial volume effect and excluded from cross-analysis in Figure 5.

**Table 2:** ^**90**^**Y dose-response of HCC portal vein tumor thrombosis by MIRD macrodosimetry**

**Patient number**	**Location**	**Approximate mean dose (Gy)**	**Follow-up (months)**	**Response within VOI**
10	Main portal vein	316	4	Complete
14	Main portal vein	248	4	Complete

**Figure 7:**
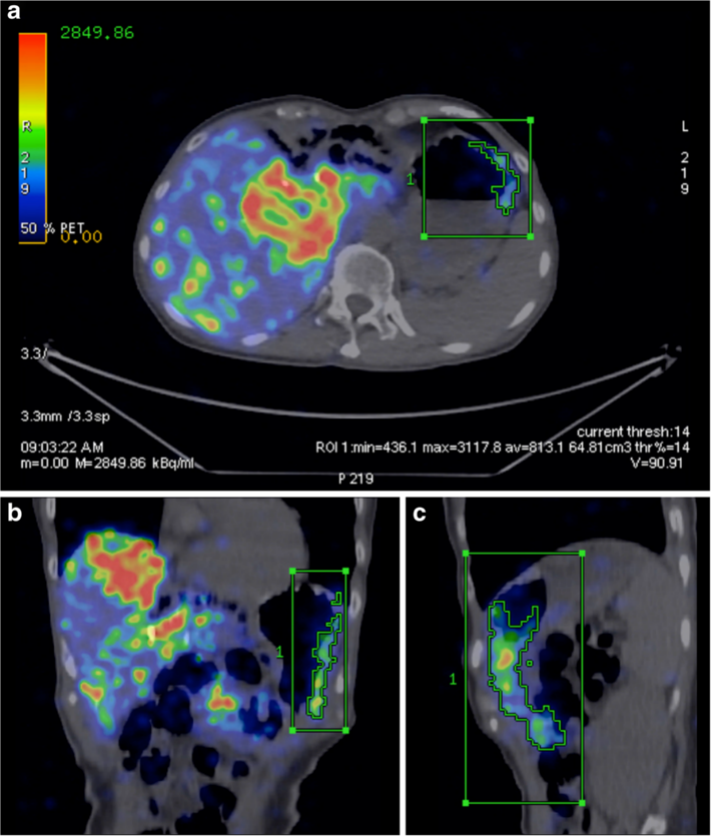
**Patient 17.** Non-target ^90^Y activity along the gastric greater curve causing CTCAE grade 3 toxicity [1]. A ^90^Y PET VOI of the activity along the gastric greater curve was defined by volumetric isocontour thresholding, visually constrained to its CT anatomical margins, shown here in **(a)** trans-axial, **(b)** coronal, and **(c)** sagittal planes. Within this VOI, the mean ^90^Y radioconcentration at the time of scan (813.1k Bq/ml) was decay-corrected back to the time of ^90^Y radioembolization (1,021.5 kBq/ml). By ^90^Y MIRD macrodosimetry, the non-target mean absorbed dose to the gastric greater curve was approximately 49 Gy within the VOI. Gastroscopy and biopsy findings were reported in part 1 [1].

All dose-response results of non-target tissue were serendipitously obtained in cases of unintended non-target shunting of ^90^Y resin microspheres due to unexpected vascular stasis and microsphere reflux during ^90^Y radioembolization. The dose-response of the stomach (Figure 7) and proximal duodenum by ^90^Y MIRD macrodosimetry are presented in Table 3. ^90^Y DVH dose-response of the right kidney is presented in Table 4. All results were in keeping with published experiences of external beam radiotherapy [29,30].

**Table 3:** ^**90**^**Y dose-response of non-target activity in viscera by MIRD macrodosimetry**

**Patient number**	**Location**	**Approximate mean dose (Gy)**	**Follow-up (months)**	**Response within VOI**
14	Gastric anterior wall	18	5.6	Asymptomatic
17	Gastric greater curve	49	3.3	Gastritis
17	Gastric pylorus	65	3.3	Ulceration
17	Proximal duodenum	53	3.3	Duodenitis

**Table 4:** ^**90**^**Y dose-response of non-target activity in the right kidney by dose-volume histograms (patient 9)**

**Site of kidney**	**Size (cm**^**3**^**)**	***V***_**20 **_**(%)**	**Mean dose (Gy)**	**Follow-up (months)**	**Response**
Right	198.3	34	18	12	Stable
Combined bilateral	381.6	8	9	As above	As above

### Accuracy of tumor predictive dosimetry

Within the statistical limitations of our small data set of HCCs treated under near-ideal dosimetric conditions, the Bland-Altman plot of intended tumor mean doses versus post-radioembolization doses by ^90^Y PET showed excellent correlation (ICC = 97.68%; Additional file 1: Figure S3). Tumor ^99m^Tc MAA SPECT/CT predictive dosimetry had a low median relative error of +3.8% (mean +6.0%, 95% CI -1.2% to +13.2%; Additional file 1: Figure S4) compared to ^90^Y PET. There may be a tendency for tumor predictive dosimetry to slightly overestimate the post-radioembolization tumor mean dose. There was no correlation between relative errors and mean T/N ratios (*r* = 0.05). Results are summarized in Additional file 1: Table S1.

## Discussion

With its superior image resolution and quantitative capability, ^90^Y PET/CT is a powerful tool to be integrated into the ^90^Y radioembolization workflow. Quantitatively, as bland microembolization by ^90^Y resin microspheres has negligible biological effect [33], clinical outcomes or toxicities in both target and non-target tissue may be predicted from dose-response radiobiology quantified by ^90^Y PET, guiding appropriate adjuvant or mitigative action.

We have reported some of the earliest ^90^Y dose-response results for resin microspheres expressed in the form of DVHs. Our threshold of *D*_70_ > 100 Gy for HCC complete response is consistent with post-radioembolization DVH case examples of HCC and melanoma liver metastasis shown by Strigari and D’Arienzo et al., respectively [34,35]. ^90^Y DVHs are able to account for the heterogeneous nature of microsphere biodistribution and are scientifically superior to MIRD macrodosimetry which falsely assumes a uniform dose distribution. dose-response data from ^90^Y DVHs have the potential to guide future improvements in predictive dosimetry such as ^99m^Tc MAA SPECT/CT-based DVHs with isodose maps to plan safer and more effective ^90^Y radioembolization [36]. For example, our data suggest that HCC tumor outcomes may be optimized by escalating the injected ^90^Y activity to achieve an intended tumor dose of *D*_70_ > 100 Gy simulated by ^99m^Tc MAA SPECT/CT DVH, within safety limitations to the lungs and non-tumorous liver.

For non-target activity, we observed that a mean absorbed dose of 18 Gy to the stomach was asymptomatic, while ≥49 Gy to the stomach or duodenum led to CTCAE grade 3 toxicity. Quantification of non-target absorbed doses by ^90^Y PET effectively translates subjective, qualitative information into objective and radiobiologically meaningful data to guide post-radioembolization care. This may involve close follow-up, extended use of proton pump inhibitors, surveillance endoscopy, or a trial of radioprotecting agents. For the kidney, we observed that a combined bilateral mean dose of 9 Gy (*V*_20_ 8%) did not result in any clinically relevant nephrotoxicity. This finding has clinical implications for the planning of safe kidney-directed ^90^Y radioembolization for renal malignancies [37,38]. We were unable to define the exact normal tissue radiation thresholds based on our limited case series. However, greater clarity on this issue shall be gained in the years ahead as post-radioembolization ^90^Y PET/CT gains popularity around the world.

In this retrospective report, we have shown tumor ^99m^Tc MAA SPECT/CT predictive dosimetry to be feasible within a small, highly select data set. Prospective validation trials on tumor predictive dosimetry are now needed, using ^90^Y PET-based absorbed doses as a reference. Our tumor dose-response results are only orientating and should be further validated by greater patient numbers and by using more adequate tumor response end points. It should also be noted that our dose-response results for both target and non-target tissue may not necessarily be valid for ^90^Y glass microspheres due to different physical characteristics.

We did not perform absorbed dose quantification of the non-tumorous liver or lungs as these are regions of much lower ^90^Y radioconcentration which may require further research and technical considerations on its quantitative accuracy by ^90^Y PET [39]. However, Elschot et al. have recently shown that ^90^Y PET-based DVHs of the non-tumorous liver may be feasible [40]. To date, there is no published data on the use of ^90^Y PET for post-radioembolization lung dose quantification. Nevertheless, we recognize the importance of tissue radiobiology in non-tumorous liver and lungs for the safe and effective clinical application of predictive dosimetry in ^90^Y radioembolization. This should be explored in future ^90^Y PET-based dosimetry research.

The specific aim of our tumor analysis was to evaluate the best possible performance of tumor predictive dosimetry planned by ^99m^Tc MAA SPECT/CT. We are therefore limited by our study design to restrict our discussion only to the tumor aspect of predictive dosimetry; our study cannot draw conclusions on predictive dosimetry applied to non-tumorous liver or lungs. To this end, we have shown in our highly select tumor data set that the intended tumor mean doses by ^99m^Tc MAA SPECT/CT predictive dosimetry correlated well with post-radioembolization doses by ^90^Y PET. From a clinical perspective, this means that if tumor predictive dosimetry had been applied to a technically successful ^90^Y radioembolization under near-ideal dosimetric conditions, then the intended tumor mean doses may be assumed valid and retrospective tumor dose quantification is usually unnecessary, unless for research or quality assurance.

In less ideal dosimetric conditions, tumor predictive dosimetry may be of variable accuracy, and retrospective tumor dose quantification by ^90^Y PET may be an option at the discretion of the nuclear medicine physician, e.g., MIRD-based predictive dosimetry applied to massive tumors with large heterogeneous areas of relative hypovascularity, where its assumption of a uniform activity biodistribution risks dosimetric uncertainty; or MIRD-based tumor predictive dosimetry applied to intended radiomicrosphere lobectomy or segmentectomy, where potential vascular stasis and reflux introduces dosimetric uncertainty. However, in technically *unsuccessful*^90^Y radioembolization, we feel that retrospective tumor dose quantification by ^90^Y PET may be routinely indicated because the intended tumor doses by predictive dosimetry may have become invalid. Furthermore, non-target activity may also be present. In such situations, the prognosis remains uncertain unless retrospective dose quantification is performed within appropriate regions of interest.

^90^Y PET/CT is but one component near the end of the entire planning-therapy continuum. ^90^Y PET/CT is a descriptive modality to assess post-implantation microsphere biodistribution, where all inadvertent or adverse findings have already irreversibly occurred. To the nuclear medicine physician, the component of the planning-therapy continuum with the greatest prognostic impact, but also more complex, is predictive dosimetry. In principle, ^90^Y radioembolization planned by predictive dosimetry is not subject to prognostic uncertainties experienced by semi-empirical ^90^Y activity prescription, a common practice within the medical oncology paradigm. In the modern era of personalized medicine, predictive dosimetry planned by modern tomographical techniques give nuclear medicine physicians unprecedented insight into the achievable outcomes for every ^90^Y radioembolization [6]. Technical success does not necessarily imply clinical success: the former is a qualitative assessment of microsphere biodistribution; the latter is a quantitative function of dose-response radiobiology. While interventional radiologists do their utmost to achieve technical success, it is the responsibility of the nuclear medicine physician to ensure that their efforts can be translated into clinical success by the rigorous application of predictive dosimetry.

## Conclusions

Qualitative and quantitative post-radioembolization ^90^Y PET/CT are two sides of the same coin and must be interpreted in the context of each other, never in isolation. Tumor and non-target tissue absorbed dose quantification by ^90^Y PET is accurate and yields radiobiologically meaningful dose-response information to guide adjuvant or mitigative action. Intended tumor mean doses by ^99m^Tc MAA SPECT/CT predictive dosimetry correlated well with post-radioembolization doses by ^90^Y PET. ^90^Y DVHs have the potential to guide future techniques in predictive dosimetry. Continued research into predictive dosimetry promises abundant rewards for safer and more effective ^90^Y radioembolization, especially as it expands into organs other than the liver.

## Competing interests

YHK, ASWG, KHT, and PKHC received research funding from Sirtex Medical Singapore. ASWG and PKHC received honoraria from Sirtex Medical Singapore. The other authors declare that they have no competing interests.

## Authors’ contributions

YHK, YST, GKYL, SS, AEHT, DCEN, and ASWG were involved in the study design, implementation, analysis, and manuscript preparation. JDS, JY, and DWT were involved in the study design, scan optimization, and manuscript preparation. CB, JAB, RJF, and TSTC were involved in the data analysis and manuscript preparation. PKHC was involved in the clinical care and manuscript preparation. MCB, FGI, RHGL, KHT, and BST were involved in the radioembolization, angiographic analysis, and manuscript preparation. All authors read and approved the final manuscript.

## Supplementary Material

Additional file 1 **Online resource including four additional figures (Figure S1, S2, S3, S4) and a table (Table S1).** Click here for file
