# Supplementary material for: Post-radioembolization yttrium-90 PET/CT - part 2: dose-response and tumor predictive dosimetry for resin microspheres
Source: EJNMMI Res. 2013 Jul 25;3:57. doi: 10.1186/2191-219X-3-57 (PMC3733999; doi:10.1186/2191-219X-3-57)
Supplement: Additional file 1 — Online resource including four additional figures (Figure S1, S2, S3, S4) and a table (Table S1). [file 2191-219X-3-57-S1.pdf]

ONLINE RESOURCE

TITLE

Post-radioembolization yttrium-90 PET/CT. Part 2: Dose-response and tumor predictive dosimetry for resin microspheres

FIRST AUTHOR CONTACT

Dr Yung Hsiang KAO

Department of Nuclear Medicine, Austin Hospital

Level 1, Harold Stokes Building, 145 Studley Road, Heidelberg

Melbourne, Victoria 3084, AUSTRALIA

Telephone: +61 3 94965718; Fax: +61 3 94576605

Email: yung.h.kao@gmail.com

TABLE OF CONTENTS

1. ^{90}Y PET voxel dosimetry using a simplified approach (page 2-3)
2. Online Resource Figure 1: Bland-Altman plot of total vial sediment activities quantified by ^{90}Y PET versus dose calibrator (page 4)
3. Online Resource Figure 2: Relative quantification error of ^{90}Y PET vs dose calibrator (page 5)
4. Online Resource Figure 3: Bland-Altman plot of tumor mean doses by ^{99m}Tc macroaggregated albumin SPECT/CT versus ^{90}Y PET/CT (page 6)
5. Online Resource Figure 4: Relative error of tumor mean doses by ^{99m}Tc macroaggregated albumin SPECT/CT versus ^{90}Y PET/CT (page 7)
6. Online Resource Table 1: Tumor mean doses by predictive dosimetry versus ^{90}Y PET voxel dosimetry (page 8)
7. References (page 8)

^{90}Y PET VOXEL DOSIMETRY USING A SIMPLIFIED APPROACH

Our ^{90}Y PET voxel dosimetry calculates the ^{90}Y absorbed dose distribution based on voxel mean radioconcentrations instead of using dose kernel or Monte Carlo methods. The reconstructed ^{90}Y PET voxels have a spatial resolution of approximately 10 to 12mm at full-width at half-maximum (FWHM), which results in blurring of spatial variations in activity distribution. This blurring effect may be considered to be similar to the 'averaging' of activity within volumes smaller than FWHM, i.e. ≈1cm^3. Hence, ^{90}Y radiation cross-fire and ^{90}Y beta dose spread may be taken to be similar to the blurring of activity due to the partial volume effect of PET. This point is further illustrated in the two examples below:

A. SIMILARITY OF ^{90}Y PET GAUSSIAN BLURRING TO ^{90}Y BETA DOSE KERNEL

Let the blurring of radioconcentration be approximated by Gaussian function with spread equal to FWHM = 10mm, and let us assume a ^{90}Y beta dose kernel such as that published by Prestwich et al. [1]. The spatial blurring of deposited energy will be similar to Gaussian blurring of PET, even though the spatial displacement due to PET is larger. In the figure below, the normalized Gaussian function (red line) and beta dose kernel (white line) as given by Prestwich et al. are plotted in grid of 0.1mm. The plot shows the shape of both functions to be very similar, although Gaussian blurring produces slightly larger displacements.

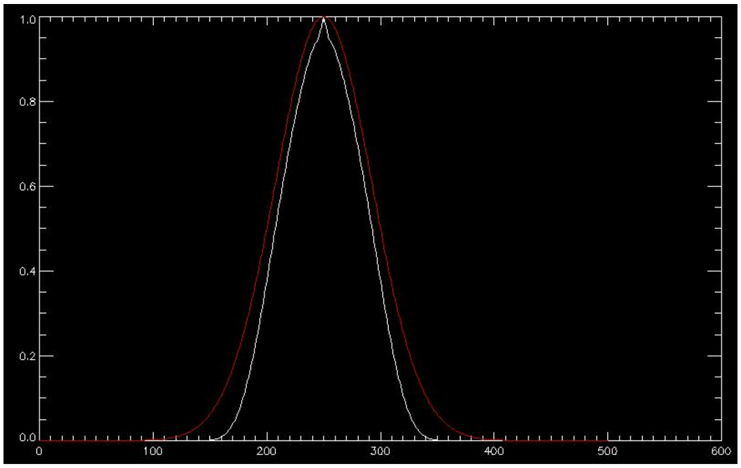

B. SMALLER ERRORS USING A SIMPLIFIED APPROACH

Lets say we have a source and background with a radioconcentration ratio of 4:1. In arbitrary units, the activity profile would be shown as the figure below:

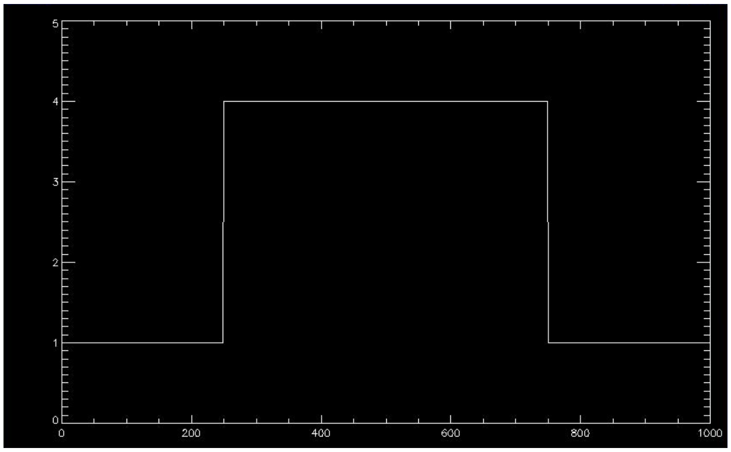

Let us assume that the Prestwich dose kernel accurately estimates the absorbed dose, and that effects of ^{90}Y bremsstrahlung and noise are negligible. Our activity measurements are provided by PET which has spatial blurring of FWHM = 10mm. However, differences from an ideal dose distribution will be smaller if we employ a simple activity profile multiplied by a dose conversion factor, rather than applying dose kernels to PET activity distribution data. Monte Carlo calculations can account for ^{90}Y bremsstrahlung and other processes, but their influence are small within therapy regions-of-interest, while its accuracy is reduced by spatial blurring of input data. The figure below shows that errors are smaller by using a simplified approach (white curve) rather than applying dose kernels (red curve).

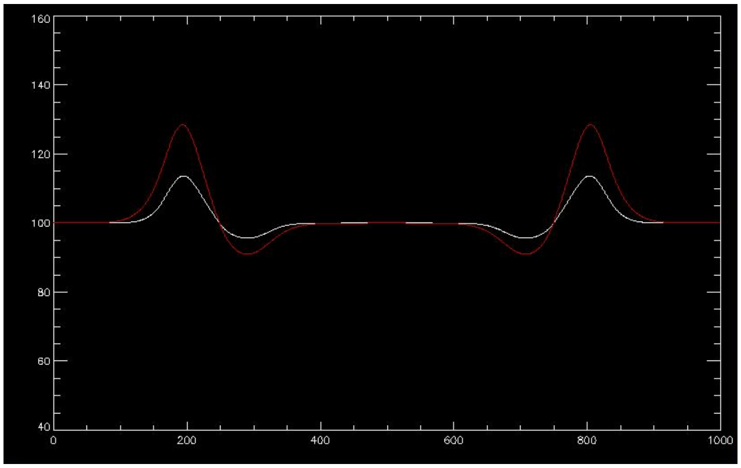

ONLINE RESOURCE FIGURE 1

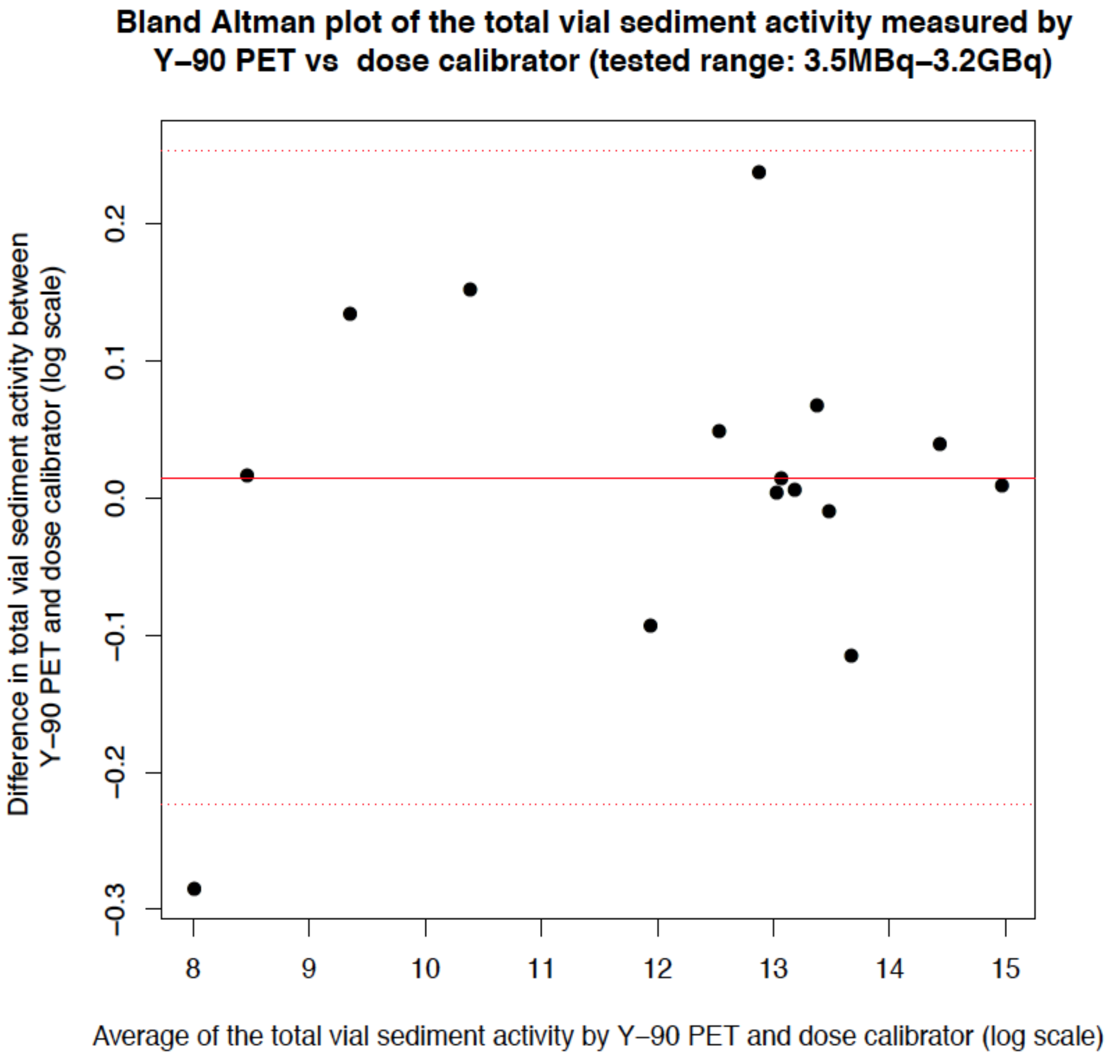

Online Resource Fig. 1 Bland-Altman plot to assess the degree of agreement between total vial sediment activities quantified by ^{90}Y PET versus a dose calibrator. There was only one value outside the limits of agreement and there appeared to be no obvious bias or patterns, indicating good reliability. The confidence limits enveloped zero and the intra-class correlation (ICC) between the two methods was above 90% (ICC = 99.85%), indicating excellent agreement

ONLINE RESOURCE FIGURE 2

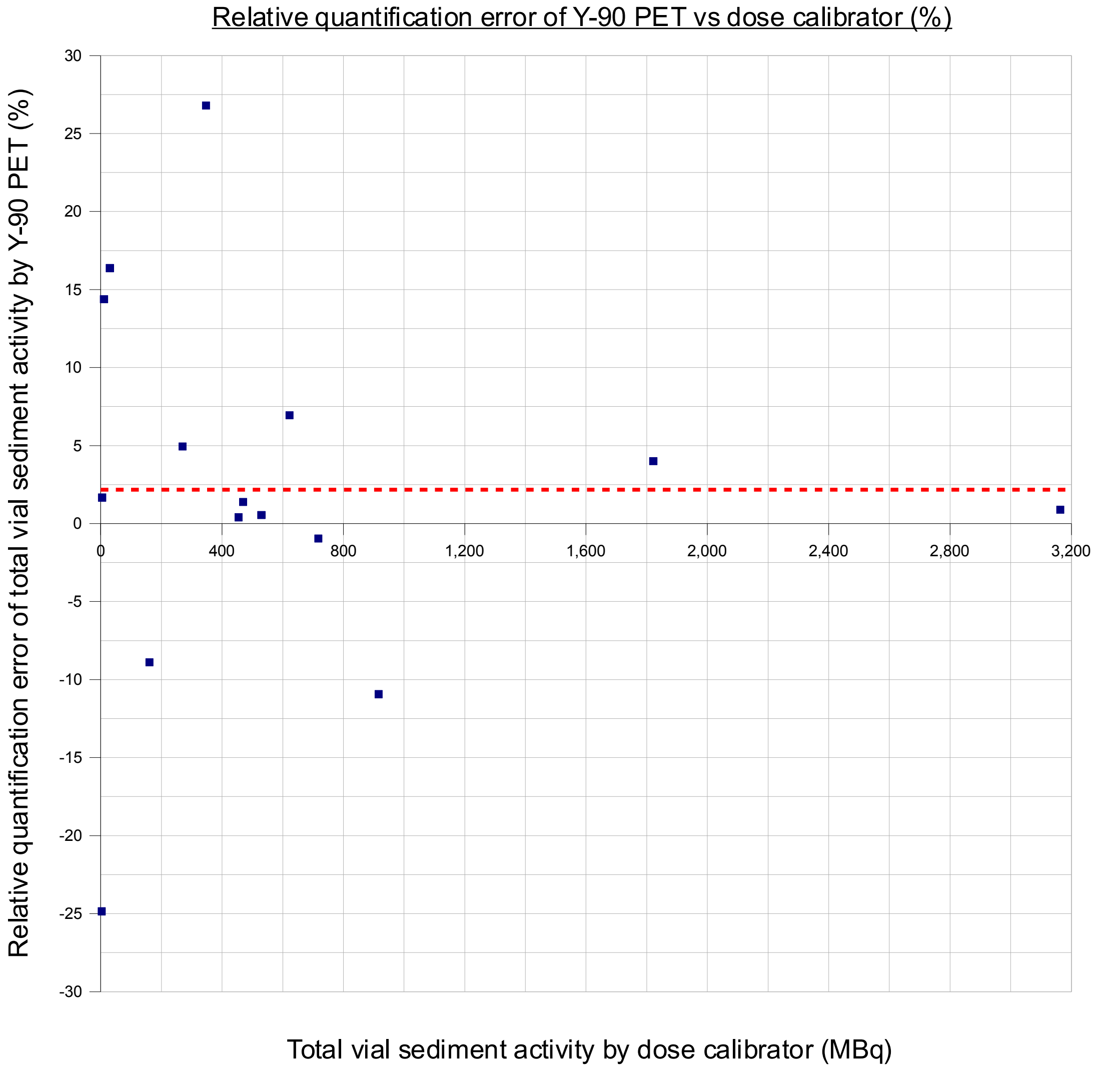

Online Resource Fig. 2 Plot of the percentage relative error of the total vial sediment activity quantified by ^{90}Y PET versus a dose calibrator. The horizontal dashed line represents the overall mean relative error

ONLINE RESOURCE FIGURE 3

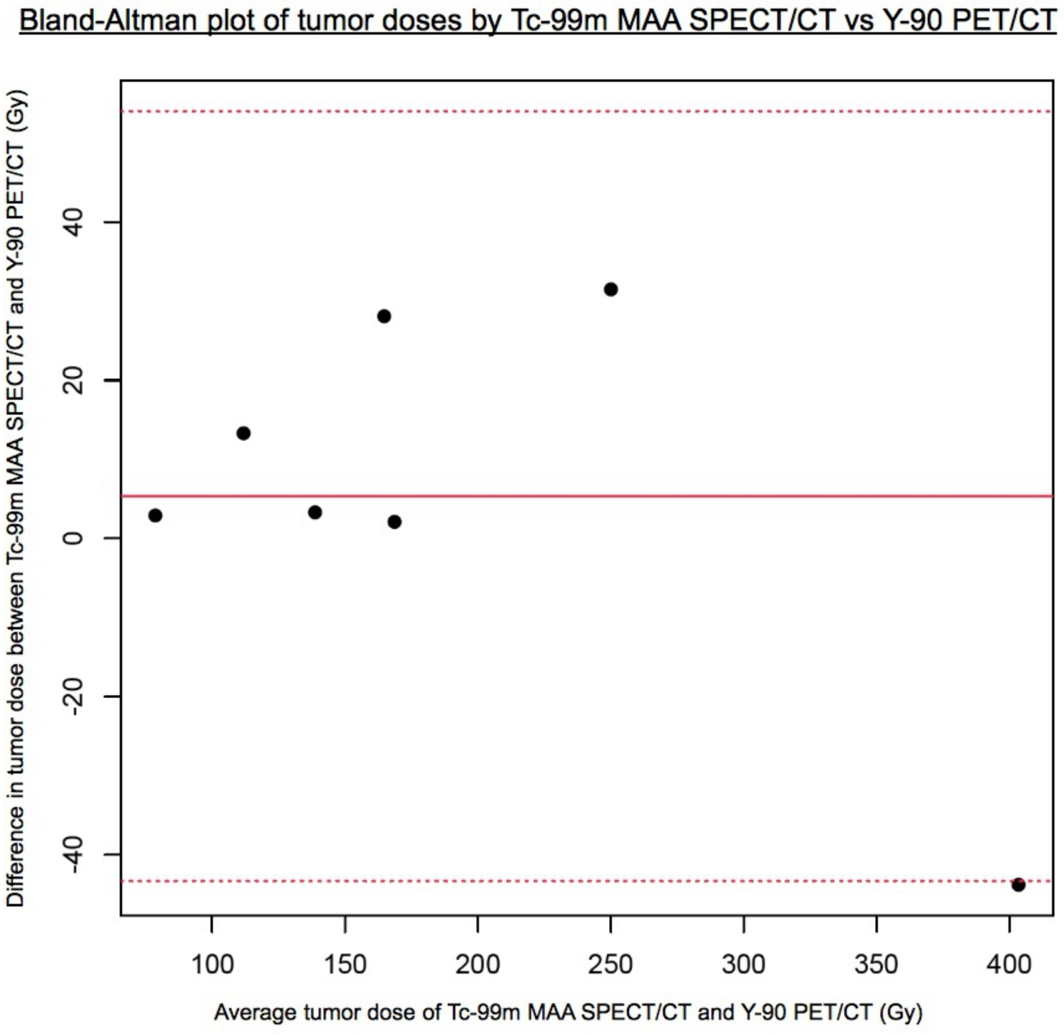

Online Resource Fig. 3 Bland-Altman plot of the intended tumor mean doses by predictive dosimetry versus post-radioembolization doses by ^{90}Y PET voxel dosimetry. These 7 data points were obtained from the same tumor dataset shown in Fig. 5 of the main report. The plot shows that the confidence limits enveloped zero and the intra-class correlation (ICC) between the two methods was above 90% (ICC = 97.68%), indicating excellent agreement

ONLINE RESOURCE FIGURE 4

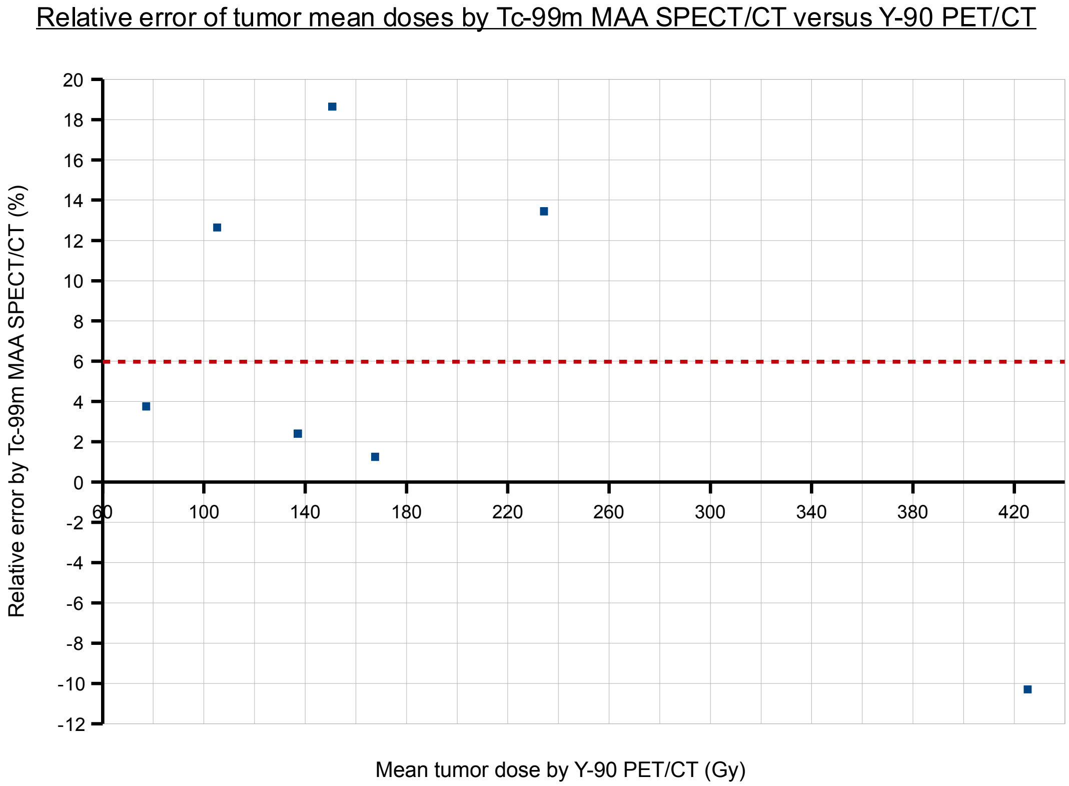

Online Resource Fig. 4 Percentage relative error of the intended tumor mean doses by predictive dosimetry versus post-radioembolization doses by ^{90}Y PET voxel dosimetry. The horizontal dashed line represents the mean relative error. These 7 data points were obtained from the same tumor dataset shown in Fig. 5 of the main report. This data is tabulated in Online Resource Table 1

ONLINE RESOURCE TABLE 1

Tumor mean doses by predictive dosimetry vs ^{90}Y PET voxel dosimetry

Patient No.	T/N ratio	Intended tumor mean dose by ^{99m}Tc MAA SPECT/CT (Gy)	Tumor mean dose by ^{90}Y PET/CT (Gy)	Relative error * (%)
1	3.7	178.8	150.7	+18.6
2	21.9	118.5	105.2	+12.6
8	3.9	169.7	167.6	+1.3
19	1.8	80.1	77.2	+3.8
21	11.8	381.5	425.3	-10.3
23	8.1 †	265.8	234.3	+13.4
23	5.0 ‡	140.4	137.1	+2.4

T/N ratio: Artery-specific mean tumor-to-normal liver ratio as estimated by ^{99m}Tc MAA SPECT/CT; * Percentage relative error of intended tumor mean doses by predictive dosimetry as compared to post-radioembolization doses by ^{90}Y PET; † Arterial territory of the left hepatic artery; ‡ Arterial territory of the right hepatic artery

REFERENCES

[1] Prestwich WV, Nunes J, Kwok CS. Beta Dose Point Kernels for Radionuclides of Potential Use in Radioimmunotherapy. J Nucl Med. 1989;30:1036-1046.
